# Rapid chain tracing of polypeptide backbones in electron-density maps

**DOI:** 10.1107/S0907444910000272

**Published:** 2010-02-12

**Authors:** Thomas C. Terwilliger

**Affiliations:** a Los Alamos National Laboratory, Los Alamos, NM 87545, USA

**Keywords:** structure solution, model building, Protein Data Bank, chain tracing, *PHENIX*, experimental electron-density maps, C^α^ positions

## Abstract

A method for the rapid tracing of polypeptide backbones has been developed. The method creates an approximate chain tracing that is useful for visual evaluation of whether a structure has been solved and for use in scoring the quality of electron-density maps. The essence of the method is to (i) sample candidate C^α^ positions at spacings of approximately 0.6 Å along ridgelines of high electron density, (ii) list all possible nonapeptides that satisfy simple geometric and density criteria using these candidate C^α^ positions, (iii) score the nonapeptides and choose the highest scoring ones, and (iv) find the longest chains that can be made by connecting nonamers. An indexing and storage scheme that allows a single calculation of most distances and density values is used to speed up the process. The method was applied to 42 density-modified electron-density maps at resolutions from 1.5 to 3.8 Å. A total of 21 428 residues in these maps were traced in 24 CPU min with an overall r.m.s.d. of 1.61 Å for C^α^ atoms compared with the known refined structures. The method appears to be suitable for rapid evaluation of electron-density map quality.

## Abstract 2

A method for rapid chain tracing of polypeptide backbones at moderate resolution is presented.

## 1. Introduction

A key step in the determination of the structure of a macromolecule by X-ray crystallography is the interpretation of the electron density in terms of an atomic model of the macromolecule. This step is important for several reasons. Firstly, it is the point at which much of the biological information can be extracted. Additionally, it is the step where confidence that the structure will be determined suddenly becomes very high. During the early stages of structure determination there will be indications that the structure may be solved, including for example a strong anomalous signal for a SAD data set, a substructure solution showing noncrystallographic symmetry, a high figure of merit of phasing or a high skew of electron density. Despite all these indications, the point where it is nearly certain that an accurate set of crystallographic phases has been obtained is when the electron density can be interpreted in terms of a model with the expected composition and geometrical features.

Model building is important in establishing confidence in a structure solution both for the benefit of the crystallographer, who can then focus on finishing the structure determination rather than obtaining more data, and for the benefit of automated procedures, which can use it as a mechanism for decision making during structure solution. If the correct hand of a heavy-atom substructure cannot be reliably identified by other methods for analysis of map quality, but the map produced using only one of the hands can be interpreted in terms of an atomic model, then that hand is much more likely to be correct than the other (see, for example, Langer *et al.*, 2008 ▶; Terwilliger *et al.*, 2009 ▶).

The speed of model building is an important factor in its utility for establishing confidence in a solution. A model-building procedure that takes hours or days to complete would normally be used to build one or a small number of models, while a procedure that takes minutes might be used more times to evaluate the effects of changing parameters and a procedure that takes seconds might be a routine approach for decision making. Additionally, a procedure that is very fast can be used effectively during X-ray data collection to make decisions about the need to collect additional data.

A number of very powerful methods for semi-automated and automated model building of proteins and nucleic acids into electron-density maps have been developed. Graphical model-building software packages such as *O* (Jones *et al.*, 1991 ▶), *MAIN* (Turk, 1992 ▶), *XtalView* (McRee, 1999 ▶) and *Coot* (Emsley & Cowtan, 2004 ▶) provide environments in which an expert user can quickly build a model into an electron-density map. These packages include tools that allow the user to define the overall locations and orientations of fragments of a model that are automatically completed by the software [*QUANTA* (Oldfield, 1994 ▶), *BATON* (Jones & Kjeldgaard, 1997 ▶), *XtalView* (McRee, 1999 ▶)] and tools to create a tracing of the paths of the polypeptide backbone and of side chains (*BONES*; Greer, 1974 ▶; Jones *et al.*, 1991 ▶).

Automated protein model-building procedures generally begin by interpreting features of the electron density to build the polypeptide backbone, followed by side-chain building. The emphasis of these methods has generally been on continually improving the quality and completeness of the models built. Some automated procedures for protein model building begin with a *BONES* tracing or identify the possible locations of C^α^ atoms and use them together with expected peptide geometries to build a polypeptide backbone [*ARP*/*wARP* (Perrakis *et al.*, 1999 ▶; Langer *et al.*, 2008 ▶), *QUANTA* (Oldfield, 1994 ▶, 2003 ▶), *CAPRA* (Ioerger & Sacchettini, 2003 ▶), *Buccaneer* (Cowtan, 2006 ▶)]. The *RAPPER* software allows a user to define the desired target features of a model and constructs models that are compatible with the available data and the target features (DePristo *et al.*, 2005 ▶). Still other software packages begin with the identification of locations of short fragments of secondary structure followed by chain extension with short fragments from a database of known structures [*MAID* (Levitt, 2001 ▶), *RESOLVE* (Terwilliger, 2003*a* ▶)] or by probabilistic consolidation of fragments (*ACMI*; DiMaio *et al.*, 2007 ▶). Recently, methods for lower resolution identification of secondary-structure elements (Baker *et al.*, 2007 ▶) and for the automatic building of double-helical nucleic acids have also been developed (Pavelcik & Schneider, 2008 ▶).

In addition to the use of automated model-building methods as stand-alone tools, these methods have been integrated into iterative procedures in which the newly built models are used to improve crystallographic phases, yielding improved maps that are in turn used for improved model building in a process that can dramatically improve the overall quality of the maps and models [*ARP/wARP* (Perrakis *et al.*, 1999 ▶; Langer *et al.*, 2008 ▶), *RESOLVE_BUILD* (Terwilliger, 2003*b* ▶), *phenix.autobuild* (Terwilliger *et al.*, 2008 ▶)].

For some time there have been parallel efforts to develop methods that assemble models by recognizing large regular features such as α-helices and β-sheets in electron-density maps [*ESSENS* (Jones & Kjeldgaard, 1997 ▶), *FFFEAR* (Cowtan, 1998 ▶, 2008 ▶)]. Recent approaches of this kind include the identification of α-helices and β-strands from density interpreted as free atoms (*ARP/wARP*; Langer *et al.*, 2008 ▶) and by the inspection of maps for the presence of tubes of density representing helices at low resolution and for pairs of nearly parallel tubes of density representing strands at higher resolution (*phenix.find_helices_strands*, Terwilliger, 2010*a*, ▶ *b* ▶). These approaches have the potential advantage that they can be used to build models into maps where the detailed features of the model (*e.g.* carbonyl O atoms, side chains) are not clearly visible, where substantial noise is present in the map and where only low-resolution maps are available. Additionally, they can potentially be faster than procedures that depend on the details of high-resolution electron density.

In this work, we focus on the speed of model building. We extend existing ideas for finding the path of a polypeptide backbone (Greer, 1974 ▶; Oldfield, 2003 ▶). We then develop a simple indexing procedure that allows the rapid construction of a C^α^ trace satisfying rudimentary geometrical and density criteria. The result is a method for building a polypeptide backbone that is fast enough to be useful as a decision-making tool during the early stages of macromolecular structure determination.

## 2. Identification of potential C^α^ positions as points along ridgelines of high density in a map

**Figure 1:**
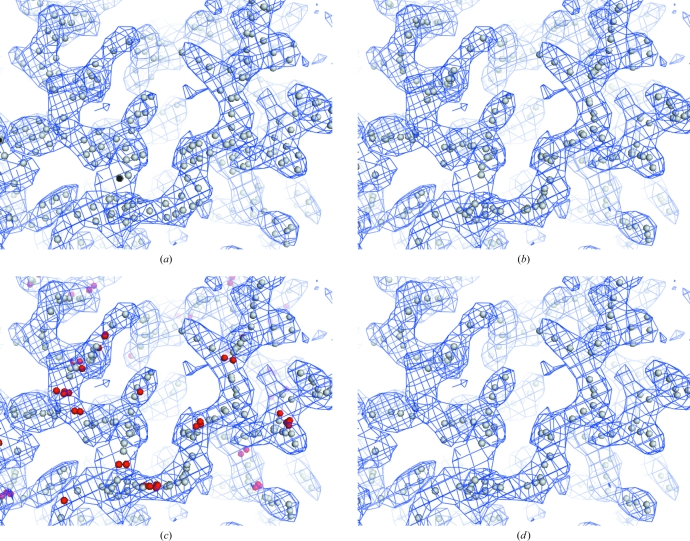
Finding potential C^α^ positions based on the density-modified electron-density map for S-hydrolase (see text). (*a*) Initial high-density points. (*b*) Points moved to the highest nearby location on the ridgeline. (*c*) Points in moderate density (in red) along lines connecting points in high density. (*d*) Potential C^α^ positions. These figures were created with *PyMOL* (DeLano, 2002 ▶).

Our method begins by finding a set of points at intervals of approximately 0.6 Å along ridgelines of high density in an electron-density map. The idea is similar to that of other ridgeline-tracing algorithms (*e.g.* Greer, 1974 ▶), with the addition of a step to adjust the coordinates of each point to be very near to the ridgeline rather than on a grid point of the map. A map is calculated, typically at a resolution of 3 Å. (If the high-resolution limit of the data is lower, the grid for the map is set as if the resolution were 3 Å.) In a first step, points near ridgelines are identified. Points on the grid used to calculate the map that are above a threshold of density (typically 1σ or higher, where σ is the r.m.s. of the map) and for which at most one of the neighboring points on the grid has a higher value are selected. The threshold of density is chosen to yield about 4*N*_total_ points, where *N*_total_ is the number of non-H atoms expected in the structure. To these points are added the highest *N*_total_/5 grid points that are at peaks in the map (with no neighbors having higher density), provided that the peaks are at least 0.5σ. This initial set of points is shown in Fig. 1 ▶(*a*) along with density-modified model-based density for the structure of S-hydrolase (PDB entry 1a7a; Berman *et al.*, 2000 ▶; Bernstein *et al.*, 1977 ▶; Turner *et al.*, 1998 ▶) obtained using the *PHENIX AutoSol* wizard with experimental MAD data (Adams *et al.*, 2002 ▶; Terwilliger, 2009 ▶).

Each of the points near ridgelines as defined above is then moved onto the ridgeline. To do this, the direction of the lowest gradient at each of these points is identified and con­sidered to be the local direction of the ridgeline. The point is then moved in the plane perpendicular to that direction to the highest nearby point accessible while continuously moving to higher density. Once all points have been moved to the nearest ridgelines, a subset of these points, separated by intervals of about 0.5 Å, is chosen using the points with the highest density values wherever possible. This set of points is shown in Fig. 1 ▶(*b*).

The points along ridgelines in Fig. 1 ▶(*b*) clearly delineate much of the path of the polypeptide backbone and of side chains in the map shown. However, there are some places in the map where there is a clear tube of density where the backbone is located but where the density is not quite high enough to be marked. We identify these places by finding pairs of points on the ridgelines that are separated by about 4 Å or less, with density all along the line between the points at least half the mean of that at the two end points. A set of points along that line, separated from each other and all existing ridgeline points by about 0.5 Å, is then added to the ridgeline points (the red points in Fig. 1 ▶ *c*).

Additionally, some points along ridgelines as defined in Fig. 1 ▶(*b*) really correspond to peaks at heavy-atom positions, disulfide positions or other nonpolypeptide-backbone positions. To reduce the number of such points (and the resulting tracing of chains through these positions), a small fraction (typically 0–0.1%) of ridgeline points with the very highest density and all points within about 3 Å of them are optionally ignored (there are none in this figure). This yields the set of points to be considered as potential C^α^ positions (Fig. 1 ▶ *d*).

### 2.1. Indexing of pairs, trimers, pentamers and nonamers of points and scoring based on geometrical and density criteria

A key step in our procedure for chain tracing is the creation of lists identifying all pairs, trimers, pentamers or nonamers of points from the list of potential C^α^ positions that satisfy basic criteria based on distances, angles and electron density. The reason for doing this is that it is then possible to carry out the calculations needed to establish whether a set of points satisfies these criteria just once. At the same time, a score is assigned to each of these pairs, trimers *etc*. that can be used later to identify which satisfy these criteria most closely.

The first of these lists is the set of all pairs of points within about 4.5 Å. This list speeds up the generation of all the other lists because the neighboring points (and their distances) have already been identified.

The second list created consists of all pairs of potential C^α^ positions that are separated by approximately 3.8 Å. This list identifies all pairs that will be considered as possible adjacent CA atoms. The range of potential C^α^—C^α^ distances that is considered is set with a tolerance *d*_target_ − *d*_tol_ to *d*_target_ + ratio_long × *d*_tol_, where the factor ratio_long is typically 0.15, so that shorter distances that are further from the target are allowed compared with longer distances; *d*_target_ is typically 3.8 Å. The value of the tolerance *d*_tol_ is used in our procedure as a way to control the number of entries in subsequent lists. For example, if too many nonamers are obtained below then the value of the tolerance *d*_tol_ can be lowered. The target number of nonamers is target_p_ratio (typically 4) times the number of expected non-H atoms in the asymmetric unit (*N*_total_).

**Figure 2:**
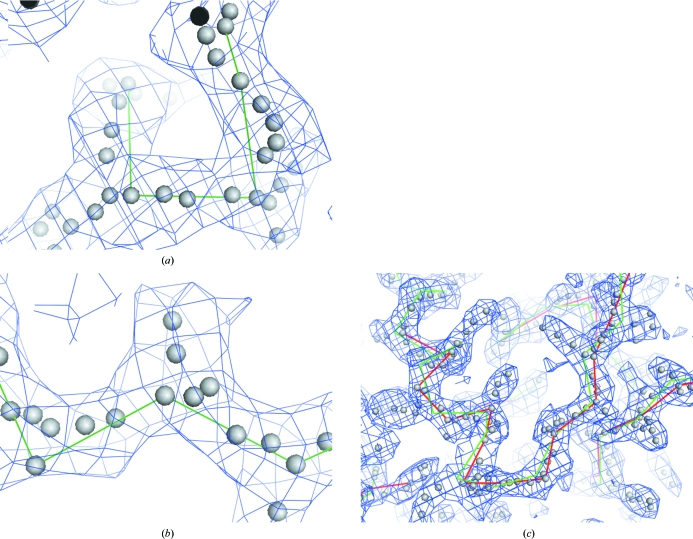
Tracing chains using potential C^α^ positions from Fig. 1 ▶ (see text). (*a*) Scoring of potential C^α^–C^α^ pairs. (*b*) Scoring of trimers. (*c*) Final connected chain (red) with refined C^α^ positions (green). These figures were created with *PyMOL* (DeLano, 2002 ▶).

Each potential C^α^–C^α^ pair is then scored based on three criteria (Fig. 2 ▶ *a*), with a lower score representing a better pair. The first criterion is the deviation between their distance and the target of 3.8 Å. The second criterion is the difference between the mean density at the potential C^α^ positions and that at the midpoint between them, divided by the mean density at the potential C^α^ positions. The rationale for this is that two points are unlikely to be adjacent C^α^ positions if the density halfway between them is very low. The third criterion is the r.m.s.d. from the line connecting the two C^α^ positions of other potential C^α^ points that are between the two C^α^ positions being considered. The rationale for this is that the density connecting adjacent C^α^ positions will normally be marked by a series of potential C^α^ positions in our method (as in Figs. 1 ▶ *b* or 1 ▶ *d*) and if the connection is a simple tube of density then all these points would generally be along the line connecting the two adjacent C^α^ positions (Fig. 2 ▶ *a*). This third criterion is scored based on the r.m.s.d. from the line con­necting the two C^α^ positions of those points that are within about 4.5 Å of one of the C^α^ positions and that are between the two C^α^ positions. In this process any points that are more than typically 2 Å from the line are given a distance to the line of 2 Å so that points that are far from the line do not dominate the calculation of the r.m.s.d. The score for a potential C^α^–C^α^ pair is simply the weighted sum of the scores from the three criteria, where the typical weighting factors are unity for the first and third criteria and 24 for the second criterion.

The third list is a list of all possible trimers, or sets of three potential C^α^ positions, that are composed of two pairs of potential C^α^ positions sharing a common potential C^α^ position and that subtend an angle typically within the range 70–180°. This allowed set of angles corresponds to the typical range of angles for sets of three sequential C^α^ atoms in a polypeptide, including a substantial tolerance for coordinate errors that are inherent in our method of choosing potential C^α^ positions. These trimers are scored based (Fig. 2 ▶ *b*) on (i) the scores of the two included pairs of potential C^α^ positions, (ii) the closeness of the angle subtended by the trimer to 110° (an approximate average for polypeptides) and (iii) the presence of a set of potential C^α^ points extending from the vertex of the trimer in the plane of the trimer in the direction away from the two ends of the trimer (approximately in the direction in which a side chain would point). The weights on these three scores are typically unity for (i), unity for (iii) (*i.e.* a score of 1 for an r.m.s.d. of 1 Å) and 1/30 for (ii) (*i.e.* a score of 1 for a 30° deviation from 110°).

The next two lists that are created are lists of all possible pentamers that can be formed from two trimers that share a common end point and of all possible nonamers formed from two pentamers that share a common end point. The pentamers and nonamers are scored by summing the values of their components and then subtracting any scores that were duplicated (*e.g.* the score of a pentamer is the sum of the scores of the three trimers it contains, less the sum of the scores of the two central pairs which are each represented in two of these trimers). In this process, any pentamers or nonamers that use any potential C^α^ positions more than once are rejected. Additionally, any pentamers or nonamers in which any pair of atoms that are not adjacent are within 4.5 Å of each other are rejected. Identification of these rejected groups is very rapid because it consists simply of identifying whether any two non-neighboring atoms in the pentamer or nonamer share any atoms in their lists of atoms located that are within a radius of 4.5 Å.

As all the components of a nonamer have previously been calculated, the creation of a list of all possible nonamers satisfying basic geometrical and density-based criteria is rapid. In the implementation discussed here, these criteria are quite rudimentary (C^α^—C^α^ distances within *d*_tol_ of 3.8 Å; C^α^—C^α^—C^α^ angles between 70° and 180°). Our scoring criteria are slightly broader but still do not include extensive geometrical criteria. Additional scoring factors such as C^α^—C^α^—C^α^—C^α^ torsion angles or end-to-end distances could be included as well using a similar framework, although they would require some additional computation using the coordinates of the C^α^ positions in the pentamers.

To speed up the next steps, the list of all possible nonamers is typically trimmed by grouping them based on the identity of the potential C^α^ atom at the center of the nonamer and then choosing only the best-scoring nonamer from each group.

### 2.2. Linking nonamers to create chains with maximal length

A second key step in our procedure is the use of a simple message-passing approach to identify for each nonamer the longest possible chain that can be created by linking it to other nonamers. In this process a specified number of overlapping C^α^ positions are allowed at the ends of linked nonamers (always at least one and typically three).

**Figure 3:**
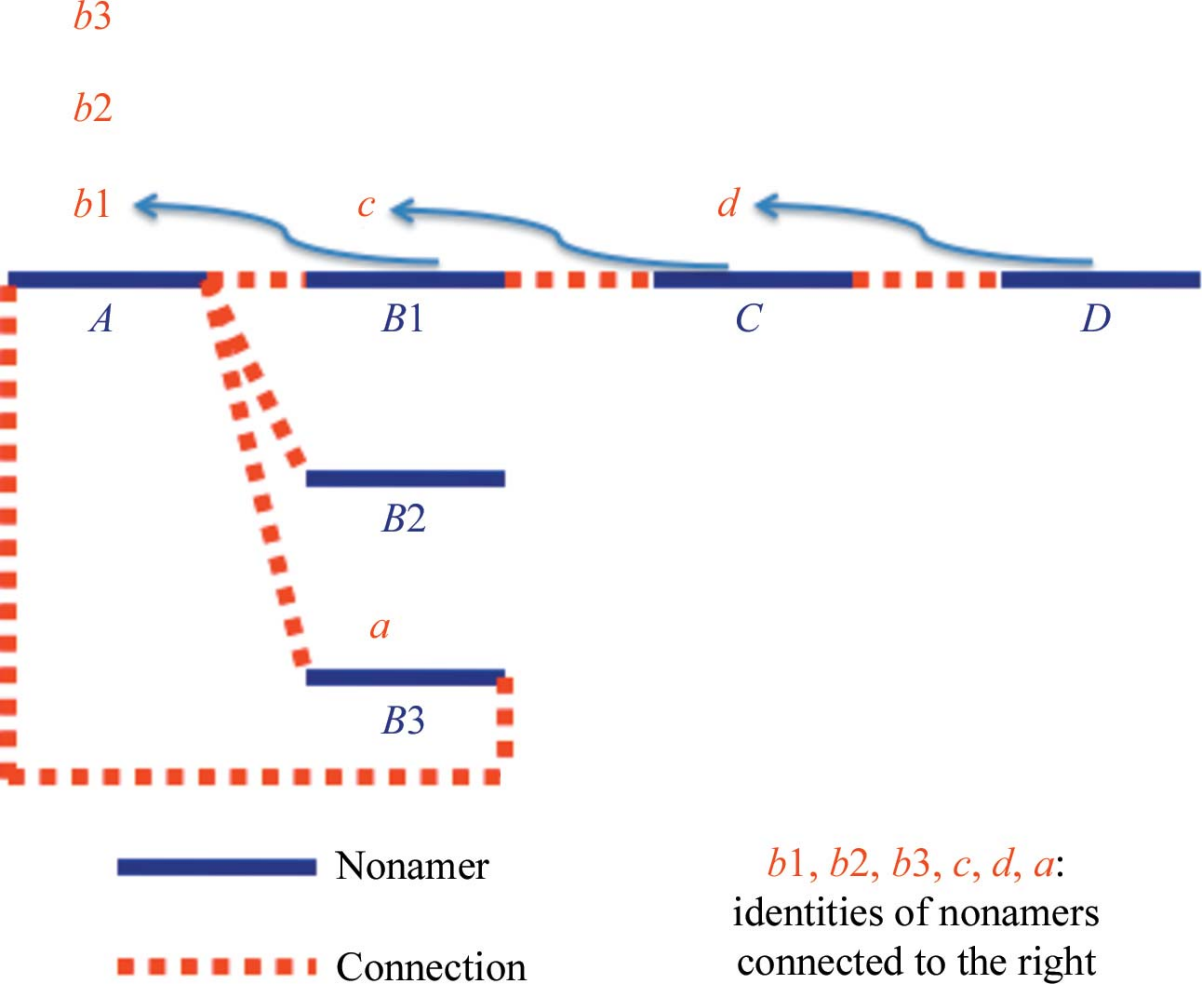
Schematic of the message-passing technique. The blue lines represent nonamers and the dotted red lines indicate connections, so that nonamer *A* is connected on the right to nonamers *B*1, *B*2 and *B*3. In the first stage of message passing, each nonamer receives, from each nonamer connected to its right, the identity of that nonamer (*e.g.* nonamer *C* receives the identity ‘*d*’ from nonamer *D*). In subsequent iterations, each nonamer receives, from each nonamer to the right, the identity (if any) that it has been passed from its connection to the right (*e.g.* nonamer *B*1 receives from *C* the identity ‘*d*’ in the second cycle and nonamer *A* receives from *B*1 the identity ‘*d*’ in the third cycle). The process is complete when no further messages are received. If a nonamer receives its own identity then the connection is ignored (*e.g.* nonamer *A* receives from nonaner *B*3 the identity ‘*a*’ in the second cycle so this circular reference is ignored).

The message-passing approach is illustrated in Fig. 3 ▶. Firstly, all pairs of nonamers that can be linked are identified, along with which end of each nonamer is involved in each such potential link. In the first cycle of message passing each nonamer passes to the left the identity of the nonamer (if any) that it is linked to on the right. (A corresponding process is carried out in the other direction but will be ignored here for clarity.) In the next cycles, each nonamer passes to the left the message that it received from the right (if any) in the previous cycle. Each nonamer also remembers the last nonamer from which it has received a message from the right. This continues until the nonamer at the far left receives a message naming the nonamer that is at the far-right end of the chain. If there are multiple possible chains involving the nomamer at the far left, the nonamer at the far left will receive a message naming the nonamer at the far-right end of the chain that is longest. At this point all the members of the chain will have remembered the identity of the nonamer to their right in this chain as well. Consequently, building up the entire longest possible chain from these messages is rapid and simple.

In this process it is possible for a set of nonamers to form a circular set of connections, so that a particular nonamer is eventually connected to itself. In these cases the message-passing procedure will lead to a nonamer eventually being passed its own identity. In our procedure we note when this happens and eliminate all chains that contain such a circular reference.

### 2.3. Choosing a set of the longest chains, removing overlaps and connection of chains

Once the longest chain containing each nonamer has been identified, a non-overlapping set of these is chosen in a hierarchical fashion. Firstly, the very longest chain is picked. All other chains that have any potential C^α^ positions overlapping (within 4.5 Å) any C^α^ position in this chain are then trimmed (or broken, as appropriate) to remove these overlapping positions. The next-longest remaining chain is then chosen and the process is repeated until there are no more chains with at least (typically) five potential C^α^ positions. This yields a possible C^α^ tracing for the macromolecule.

Chains of C^α^ atoms obtained in this way can sometimes end near the beginning of another chain but not be connected, if no nonamer was present that could link the two chains. Trimers and pentamers of C^α^ atoms were used to fill in some of these gaps. Once a single set of non-overlapping chains was obtained as described above, each pair of ends of these chains was examined to determine whether the ends could be connected using a trimer or pentamer of C^α^ atoms. If so, the longest chains that could be obtained in this way were chosen and a new non-overlapping set of chains was identified. In making these connections, the requirement that the connecting trimers or pentamers share the C^α^ atoms present at the ends of the chains was relaxed. Instead, C^α^ atoms in these connecting trimers or pentamers had to be within a specified distance (typically 1 Å) of a C^α^ position at the end of a chain to be connected, allowing a greater number of chains to be connected. Fig. 2 ▶(*c*) shows the final connected chains obtained in the region shown for the examples in Fig. 1 ▶.

### 2.4. Identification of helices and strands within chains and scoring of secondary-structure elements

The C^α^ traces that are obtained from the procedures described above are non-directional; they could equally well have their N- or C-termini at a particular end of the chain. To help identify the direction of the chains, we carried out a simple distance-based procedure to identify α-helices and β-­strands in these chains. A set of six or more sequential C^α^ positions was considered to be α-helical if the C^α^ positions separated by three residues (each *i*→*i* + 3 distance) was 5.5 ± 1.25 Å and if the C^α^ positions could be matched to those of an idealized α-helix within a tolerance of typically 1.5 Å. Similarly, a set of five or more C^α^ positions was considered to be a β-strand if C^α^ positions separated by three residues were 10.5 ± 1.25 Å apart. We then used the procedures that we have recently developed for the identification of helix and strand directions (*phenix.find_helices_strands*; Terwilliger, 2010*a*, ▶ *b* ▶) to tentatively assign chain direction to each strand or helix segment in a chain. If all the directions of all the helices and strands within a chain were the same, then the chain was assigned that direction. Otherwise, the chain direction was considered to be unknown.

The secondary structure in the C^α^ trace obtained using this procedure was scored with a simple algorithm in which the number of residues identified above as being α-helical was added to the number of residues in paired β-strands. Paired β-­strands were simply those β-strands that were approximately 4.5 ± 2.0 Å from another strand. In order to reduce the scores of models built from maps that were inverted, any residues in β-sheets that showed a clear negative twist were ignored in this calculation. The twist of sheets was calculated from the mean rotation occurring from one pair of C^α^ atoms to the next along a pair of adjacent strands; if the mean rotation was more negative than one standard deviation of the mean this pair of strands was skipped when calculating residues in secondary structure.

### 2.5. Optional conversion from C^α^ models to all-atom models with *PULCHRA* and chain assembly with *RESOLVE*

The C^α^ models obtained above were optionally converted to polyglycine models using *PULCHRA* (Rotkiewicz & Skolnick, 2008 ▶), a procedure that uses distance criteria and a database of common conformations to identify backbone polypeptide conformations. In cases where the chain direction was not known, both chain directions were used.

A final optional step in the procedure is to use the chain-assembly procedures in *RESOLVE* (Terwilliger, 2003*a* ▶) to remove overlapping segments of chains, to identify the chain direction and to create a single polyglycine model (with chain breaks). The *RESOLVE* assembly procedure scores chains based on the density at the coordinates of main-chain atoms. Consequently, in cases where both directions of a chain are included in the assembly process the chain direction that yields the higher score is included. The *RESOLVE* assembly procedure can include any number of starting fragments, so that in cases where α-helices and β-strands have been identified prior to chain tracing the fragments from those searches can also be included.

## 3. Application to density-modified experimental electron-density maps

We tested the chain-tracing algorithm described above on a set of 42 density-modified electron-density maps produced by the *PHENIX AutoSol* wizard (Terwilliger *et al.*, 2009 ▶) using experimental MAD, SAD and MIR data (Table 1 ▶). Each map was calculated at a resolution of 3 Å for the chain-tracing procedure. These density-modified 3 Å maps had a range of quality; their correlation with maps based on the corresponding refined structures varied from 0.47 to 0.84. The refined structures represented by the 42 maps contained a total of 26 651 residues. The chain-tracing algorithm con­structed chains with a total of 21 428 residues (80%), with an overall r.m.s.d. between C^α^ atoms in the models and those in the refined structures of 1.61 Å. Overall, 46% of the C^α^ atoms in the models were in secondary structure (α-helix or β-­sheets). The total CPU time required to build these models (using 2.9 GHz Intel Xeon processors) was 24 min or about 0.07 s per residue traced.

**Figure 4:**
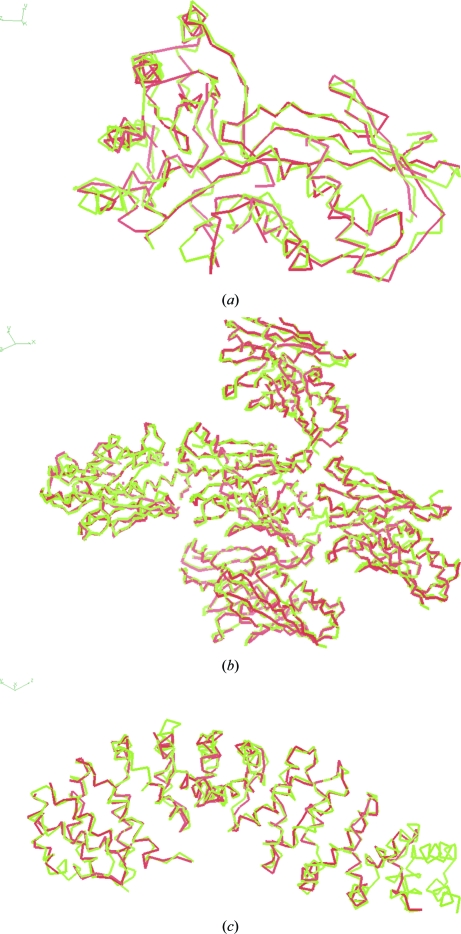
Backbone diagrams of chain tracings. (*a*) Mevalonate kinase (PDB entry 1kkh; Yang *et al.*, 2002 ▶). (*b*) Structural genomics target 1038B (PDB entry 1lql; Choi *et al.*, 2003 ▶). (*c*) Armadillo repeat region of murine β-catenin (PDB entry 3bct; Huber *et al.*, 1997 ▶). Red tracings are from the present method; green tracings are from the deposited structures. These figures were created with *Coot* (Emsley & Cowtan, 2004 ▶).

Fig. 4 ▶ shows three examples of the models produced by the chain-tracing algorithm using high-quality maps. Fig. 4 ▶(*a*) illustrates the model built for mevalonate kinase (PDB entry 1kkh; Yang *et al.*, 2002 ▶). This map had a correlation with the model map of 0.80 at a resolution of 3 Å. The model is largely complete, with 302 of 317 residues traced in 9 s of CPU time. A total of 66% of the traced chains were in identifiable secondary structure and the model is quite similar to the refined model, with an r.m.s.d. for C^α^ atoms of 1.38 Å. A second example is shown in Fig. 4 ▶(*b*), which shows a section of the model for the structural genomics target 1038B (PDB entry 1lql; Choi *et al.*, 2003 ▶). For this map, with a correlation to the model map of 0.71 at a resolution of 3 Å, 1308 of 1432 residues were traced in 114 s of CPU time, producing a structure in which 70% of the residues were in secondary structure and with an r.m.s.d. to the refined structure of 1.39 Å. A third example, shown in Fig. 4 ▶(*c*), is the armadillo repeat region of β-catenin (PDB entry 3bct; Huber *et al.*, 1997 ▶). This map had a correlation to the model map of 0.81 and 369 of 457 residues were traced in 23 s of CPU time, yielding a model with an r.m.s.d. to the refined structure of 1.21 Å and with 59% of the model in identified secondary structure.

To place the chain-tracing algorithm developed here in context, Table 2 ▶ compares this procedure with other model-building algorithms that are available in *PHENIX*. The most accurate method available is the *phenix.autobuild* procedure (Terwilliger *et al.*, 2008 ▶), which integrates *RESOLVE* model building with routines for building regions that have not yet been built and connecting chains with nearby ends and which uses *phenix.refine* refinement (Afonine *et al.*, 2005 ▶) to improve the model during the procedure. One cycle of the *phenix.autobuild* procedure (Table 2 ▶) yields models with an overall r.m.s.d. from the corresponding refined models of 0.95 Å, but takes 42 h to build 20 601 residues, a rate of just 0.1 residue per second. The *RESOLVE* model-building procedure (using the superquick build option) is about ten times faster (1.1 residues per second) and yields a similar number of residues (19 037), but the r.m.s.d. is higher (1.16 Å). Using methods for finding α-­helices and β-strands in density maps (the *phenix.find_helices_strands* algorithms; Terwilliger, 2010*a*, ▶ *b* ▶), a smaller number of residues in secondary structure can be found (12 322) with a slightly poorer r.m.s.d. (1.24 Å), but the procedure is faster (2.3 residues per second). Finally, the current chain-tracing method gives about as many residues (21 428) as *phenix.autobuild* and is much faster (15 residues per second), but has a higher r.m.s.d. (1.61 Å).

As the chain-tracing procedure described here is rapid and yields estimates of the secondary-structure content of the structures, it seemed possible that the approach could be used for both visual and automated analyses of the quality of electron-density maps. In essence, the secondary-structure content of the model might be a useful indicator of whether the structure is ‘solved’ or close to being solved.

**Figure 5:**
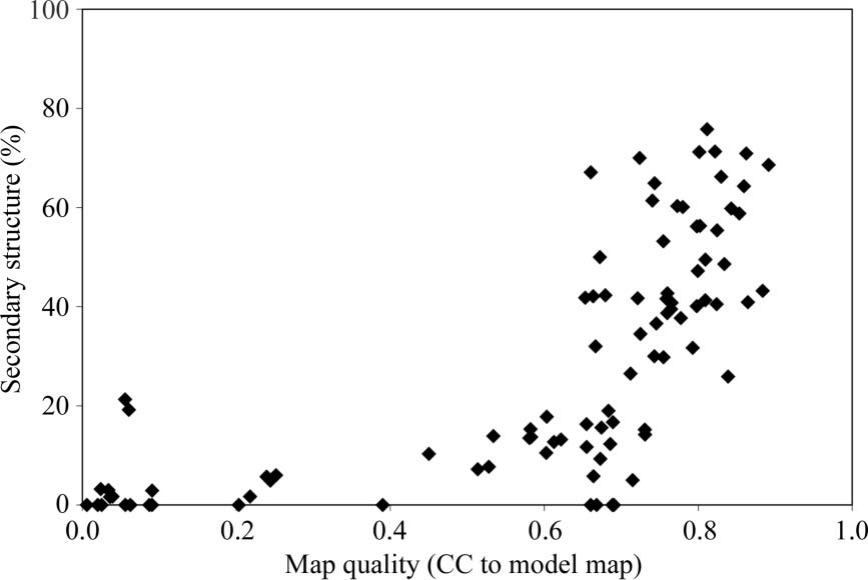
Secondary structure identified in models compared with map quality (see text). Map quality is the correlation of the map with one based on the refined structure. Secondary structure is the percentage of residues in α-­helices or β-sheets in these models identified as described in the text.

We examined the use of chain tracing as a quality indicator by applying the algorithm to 92 density-modified electron-density maps that were created during *PHENIX AutoSol* wizard structure solution of the 42 structures listed in Table 1 ▶. The *AutoSol* wizard creates density-modified maps for those experimental maps that either have the highest scores in the density-modification procedure or that have scores that are within about two standard deviations of those highest scores so that they cannot clearly be ruled out. These typically include the opposite hand of the heavy-atom substructures for MIR structures. Fig. 5 ▶ plots the percentage of residues identified as being within secondary structure as a function of the correlation between the density-modified maps used in the tracing and the maps based on the corresponding refined structures. Fig. 5 ▶ shows that maps that yield a model with a secondary-structure percentage of about 10% or greater are very likely to have a high correlation (0.6 or greater) with the map based on the refined model of the structure. A cutoff of 10% secondary structure in this evaluation procedure misses some maps of high quality (there are a few maps in Fig. 5 ▶ with a secondary-structure percentage of about 5–10% but high map quality), but it appears to be a generally useful criterion.

There are two maps indicated in Fig. 5 ▶ that had very low correlations to model maps yet yielded moderate percentages of secondary structure in the models. The two points at the left of the figure with map-correlation values of 0.06 and secondary-structure percentages of about 20% are MIR maps for RNAse S (PDB entry 1rge; Sevcik *et al.*, 1996 ▶) in which the hands of the heavy-atom sites are inverted. The resulting maps are inverted but are otherwise partly or completely traceable. In each case the twist of the β-sheets in the models that were built was negative (as expected for an inverted map). The value of the twist was not certain in each case, however, so that according to our procedure the residues in these sheets were still included in the count of residues in secondary structure (the values of twist were −8 ± 8° and −14 ± 20° per residue for the two models). The structure also has only about 15% α-­helical structure, so the inversion of the maps was difficult to identify automatically. Considering that these two maps have some real (though inverted) features of polypeptide chains, Fig. 5 ▶ indicates that the secondary-structure content in a chain-tracing model built from an electron-density map can be quite a good indicator of the overall quality of the map.

**Figure 6:**
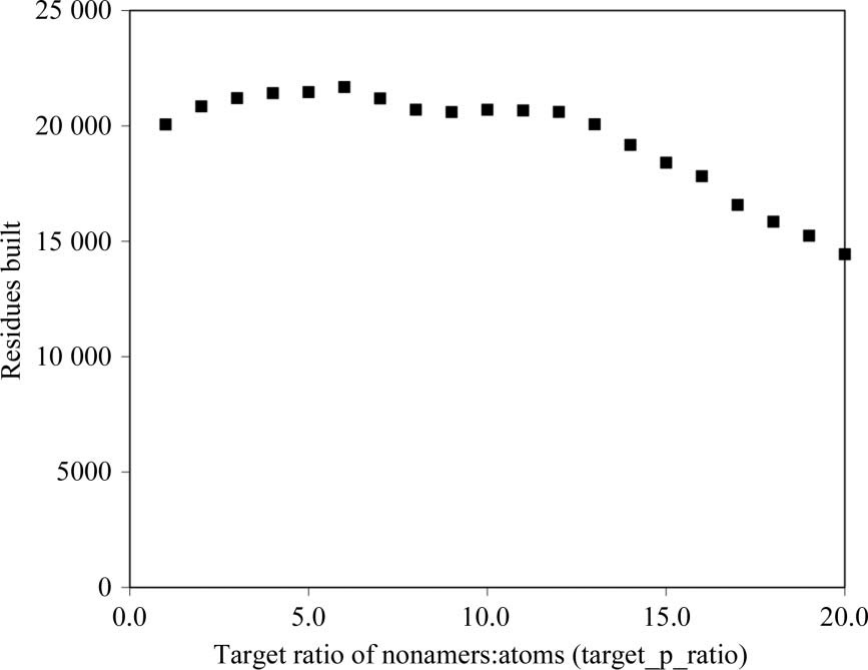
Number of residues built as function of the target ratio of nonamers to atoms.

One adjustable parameter in this procedure is the target ratio of the number of nonamers to identify to the number of non-H atoms expected in the structure (target_p_ratio). This parameter is used to adjust the tolerance of C^α^—C^α^ distances, thereby adjusting the number of potential pairs, trimers, pentamers and nonamers to be considered. In an ideal situation a large number of nonamers would be considered; how­ever, in a practical application both the time required for the calculations and the memory usage increases with the number of nonamers and in particular with the number of links that connect nonamers. With a fixed size of the arrays used to store links between nonamers, if more memory is required than is available then some of the links are simply ignored. Fig. 6 ▶ illustrates this compromise for the 42 maps in Table 1 ▶ using the ‘huge’ version of *RESOLVE* with a maximum of 10^7^ links between nonamers. Increasing target_p_ratio from 1 to 6 leads to an increase in the total number of residues built, but further increases in target_p_ratio reduce the number built. Over the entire range shown, the overall r.m.s.d. between C^α^ positions obtained with the chain tracing and those of the refined models was relatively constant, varying from 1.57 to 1.63 Å. The default value of target_p_ratio = 4 appears to be a reasonable compromise, although in individual cases a larger number of residues built could be obtained by using a version of *RESOLVE* with larger arrays.

## 4. Conclusions

The chain-tracing procedure described here is quite rapid and can give relatively complete tracings of polypeptide chains for electron-density maps of high quality. An analysis of the secondary structure in the models that are produced can produce a good indication that the map is largely correct. As the procedure is quite rapid, it can be a useful tool for visual inspection of the quality of a map as well as a part of automated analyses of electron-density maps.

## Figures and Tables

**Table 1:** Chain-tracing in experimental electron-density maps

Structure	*d*_min_ (Å)	Map quality (CC to model map using data to 3 Å)	Residues	Residues built	C^α^ r.m.s.d. (Å)	Residues in secondary structure (%)	CPU time (s)
RNase P (1nz0; Kazantsev *et al.*, 2003 ▶)	1.5	0.53	416	284	2.53	8	14
1063B (1lfp; Shin *et al.*, 2002 ▶)	1.7	0.68	243	132	1.98	17	7
Epsin (1edu; Hyman *et al.*, 2000 ▶)	1.8	0.89	149	132	1.31	43	6
Isocitrate lyase (1f61; Sharma *et al.*, 2000 ▶)	1.8	0.65	836	754	1.59	42	81
MBP (1ytt; Burling *et al.*, 1996 ▶)	1.8	0.89	227	194	1.41	69	9
P9 (1bkb; Peat *et al.*, 1998 ▶)	1.8	0.81	136	128	1.61	76	9
Penicillopepsin (3app; James & Sielecki, 1983 ▶)	1.8	0.84	323	279	1.58	41	10
Myoglobin (Ana Gonzales, personal communication)	1.9	0.73	154	139	1.96	5	10
ROP (1f4n; Willis *et al.*, 2000 ▶)	1.9	0.84	108	107	2.07	60	4
1167B (1s12; Shin *et al.*, 2005 ▶)	2.0	0.72	370	254	1.77	42	10
CobD (1kus; Cheong *et al.*, 2002 ▶)	2.0	0.80	355	331	1.73	32	18
NSF-N (1qcs; Yu *et al.*, 1999 ▶)	2.0	0.80	195	162	1.57	40	8
Synapsin (1auv; Esser *et al.*, 1998 ▶)	2.0	0.78	585	421	1.71	60	24
Tryparedoxin (1qk8; Alphey *et al.*, 1999 ▶)	2.0	0.79	143	142	1.67	47	6
PDZ (1kwa; Daniels *et al.*, 1998 ▶)	2.1	0.67	174	130	1.65	50	7
Fusion complex (1sfc; Sutton *et al.*, 1998 ▶)	2.3	0.73	867	643	1.98	14	141
GPATase (1ecf; Muchmore *et al.*, 1998 ▶)	2.3	0.82	992	901	1.49	71	50
Granulocyte (2gmf; Rozwarski *et al.*, 1996 ▶)	2.3	0.62	241	141	1.80	16	8
VMP (1l8w; Eicken *et al.*, 2002 ▶)	2.3	0.76	1141	833	1.42	41	37
Armadillo (3bct; Huber *et al.*, 1997 ▶)	2.4	0.86	457	369	1.21	59	23
Cyanase (1dw9; Walsh *et al.*, 2000 ▶)	2.4	0.82	1560	1506	1.71	55	62
Mev kinase (1kkh; Yang *et al.*, 2002 ▶)	2.4	0.83	317	302	1.38	66	9
NSF D2 (1nsf; Yu *et al.*, 1998 ▶)	2.4	0.84	247	243	1.59	49	11
1102B (1l2f; Shin, Nguyen *et al.*, 2003 ▶)	2.5	0.78	344	308	1.45	56	22
AEP transaminase (1m32; Chen *et al.*, 2002 ▶)	2.5	0.81	2169	2045	1.32	71	95
FLR (1bkj; Tanner *et al.*, 1996 ▶)	2.5	0.77	460	401	2.01	39	13
P32 (1p32; Jiang *et al.*, 1999 ▶)	2.5	0.86	529	475	1.38	71	13
PSD-95 (1jxm; Tavares *et al.*, 2001 ▶)	2.5	0.76	264	231	1.46	53	13
QAPRTase (1qpo; Sharma *et al.*, 1998 ▶)	2.5	0.71	1704	1209	1.53	35	69
RNase S (1rge; Sevcik *et al.*, 1996 ▶)	2.5	0.65	192	133	2.06	42	7
Gene V (1vqb; Skinner *et al.*, 1994 ▶)	2.6	0.74	86	74	1.52	65	4
Rab3A (1zbd; Ostermeier & Brünger, 1999 ▶)	2.6	0.82	301	262	1.55	41	19
GerE (1fse; Ducros *et al.*, 2001 ▶)	2.7	0.70	384	317	1.41	27	14
CP synthase (1l1e; Huang *et al.*, 2002 ▶)	2.8	0.75	534	253	1.53	40	18
Rh dehalogenase (1bn7; Newman *et al.*, 1999 ▶)	2.8	0.78	291	270	1.42	56	9
S-hydrolase (1a7a; Turner *et al.*, 1998 ▶)	2.8	0.81	861	813	1.62	41	43
UT synthase (1e8c; Gordon *et al.*, 2001 ▶)	2.8	0.78	990	867	1.53	60	48
1029B (1n0e; Chen *et al.*, 2004 ▶)	3.0	0.73	1130	1016	1.57	61	37
1038B (1lql; Choi *et al.*, 2003 ▶)	3.0	0.71	1432	1308	1.39	70	114
1071B (1nf2; Shin, Roberts *et al.*, 2003 ▶)	3.0	0.65	801	760	1.63	67	62
Synaptotagmin (1dqv; Sutton *et al.*, 1999 ▶)	3.2	0.67	275	199	2.55	19	29
GroEL (1oel; Braig *et al.*, 1995 ▶)	3.8	0.55	3668	1960	1.98	14	247

**Table 2:** Comparison of model-building procedures

Method	Residues built (of 26651 possible residues in 42 experimental density-modified maps)	R.m.s.d. (Å)	Time (s)	Residues per second
trace_chain†	21428	1.61	1441	14.9
Helices–strands‡	12322	1.24	5331	2.3
*RESOLVE*§	19037	1.16	16933	1.1
*phenix.autobuild*¶	20601	0.95	155767	0.1

† trace_chain is the method in this paper (without optional assembly steps) with *phenix.find_helices_strands* and trace_chain=True. The r.m.s.d. is for C^α^ atoms only.

‡ Helices–strands is a combination of finding α-helices and β-strands with *phenix.find_helices_strands* and trace_chain=False (Terwilliger, 2010*a*, ▶ *b* ▶).

§ *RESOLVE* is the superquick option for model building in *RESOLVE* (Terwilliger, 2003*a* ▶).

¶ *phenix.autobuild* is the standard model-building procedure in *PHENIX* and includes several cycles of model building with *RESOLVE* alternating with atomic refinement with *phenix.refine* (Afonine *et al.*, 2005 ▶).
